# Better Handgrip Strength Is Related to the Lower Prevalence of Pain and Anxiety in Community-Dwelling Older Adults

**DOI:** 10.3390/jcm12113846

**Published:** 2023-06-04

**Authors:** Natalia Sosowska, Agnieszka Guligowska, Bartłomiej Sołtysik, Ewa Borowiak, Tomasz Kostka, Joanna Kostka

**Affiliations:** 1 Department of Geriatrics, Healthy Ageing Research Centre, Medical University of Lodz, 90-419 Lodz, Poland; natalia.sosowska@umed.lodz.pl (N.S.); agnieszka.guligowska@umed.lodz.pl (A.G.); bartlomiej.soltysik@umed.lodz.pl (B.S.); tomasz.kostka@umed.lodz.pl (T.K.); 2 Department of Conservative Nursing, Medical University of Lodz, 90-419 Lodz, Poland; ewa.borowiak@umed.lodz.pl; 3 Department of Gerontology, Medical University of Lodz, 90-419 Lodz, Poland

**Keywords:** pain, anxiety, elderly, handgrip, quality of life

## Abstract

Although handgrip strength (HGS) may be treated as a biomarker of many health problems, there is little evidence on the potential role of HGS in the prevention of pain or anxiety in older adults. We investigated the relationship of HGS to the presence of pain and anxiety among community-dwelling older adults. The study was performed in 2038 outpatients, aged 60 to 106 years. The Jamar hand-held hydraulic dynamometer was used to measure HGS. The prevalence of pain and anxiety was assessed with the Euroqol 5D questionnaire. Symptoms of depression were recorded with 15-item Geriatric Depression Scale (GDS). In the multivariate logistic regression model taking into account age, sex, BMI and concomitant diseases, the significant influence of HGS on the presence of pain (odds ratio [OR]  =  0.988) in the entire study population and among men (OR  =  0.983) was found. HGS was a significant independent predictor for the presence of anxiety in the entire study population (OR  =  0.987), in women (OR  =  0.985) and in men (OR  =  0.988). In the fully adjusted model with included GDS, 1 kg higher HGS was still associated with 1.2% and 1.3% lower probability of the presence of pain and anxiety, respectively. We conclude that low HGS is associated with the presence of pain and anxiety among older adults, independent of age, sex, depression symptoms and concomitant chronic diseases. Future research should assess whether improvement of HGS would alleviate psychological dysfunction in older adults.

## 1. Introduction

Chronic pain is common among older adults and is associated with suffering, falls and disability. It is a risk factor for premature death and accelerated cognitive decline. It also causes social isolation and generates greater costs and burden on health systems [1]. Drug treatment is only partially effective and is associated with adverse effects [2]. As the number of older adults in the population increases, anxiety is also becoming a common problem, resulting in increased burden on healthcare, contributing to high social and individual costs. Late detection of this disorder is associated with uncharacteristic symptoms, multimorbidity and the ageing process itself [3]. Therefore, more research should be implemented to improve the diagnosis of pain and anxiety in the elderly and explore their relationship with other factors, which may contribute to better prevention, influence the way these complaints are approached and improve the quality of treatment. 

One of the potential simple measures related to pain and anxiety may be handgrip strength (HGS) [4,5]. Because of greater muscle mass, males are on average stronger than females [6]. HGS may be treated as a biomarker, as it can be used to determine overall strength, upper limb function, sarcopenia, frailty syndrome, bone mineral density, fractures, falls, malnutrition, cognitive impairment, depression, sleep problems, diabetes, multimorbidity and quality of life at the same time [4,7,8,9,10]. Monitoring and sustaining HGS can act as a preventive measure of mortality and the appearance of diseases, functional decline and hospitalization problems [11,12,13,14]. Routine use of HGS can be as a single measurement or as part of several investigations to identify older adults who may present with poor health [4].

In the available literature, there are some data on the impact of HGS on pain or anxiety in specific populations of patients [15,16,17,18,19]. Nevertheless, there is a paucity of data on the potential role of sustaining HGS in the prevention of feelings of pain or anxiety in older adults, especially in larger populations of seniors. Therefore, in this work, we investigated the relationship of HGS to the presence of pain and anxiety among community-dwelling older adults. 

## 2. Materials and Methods

The study was performed in 2038 outpatients of the Geriatric Clinic of the Medical University of Lodz, Poland, aged 60 to 106 years who volunteered to participate in the study. The inclusion criteria were age 60 years and over, living in the community, in-person contact that allows them to understand the instructions logically and written consent to participate in the study. We excluded patients who were not able or refused to perform the necessary tests and who had recently undergone hand surgery or treated for inflammation at this location. 

The study was approved by the Bioethics Committee of the Medical University of Lodz and complies with the Declaration of Helsinki and Good Clinical Practice Guidelines.

### 2.1. Euroqol 5D Quality of Life Assessment Questionnaire

The Euroqol 5D questionnaire is a widely used and validated generic instrument [20,21]. The test is divided into two parts. The first consists of five statements relating to mobility, self-care, usual activities, pain/discomfort and anxiety/depression. One can choose from ‘no problems’, ‘moderate problems’ and ‘extreme problems’. The second part is a visual analogue scale or Euroqol-VAS and is a ‘thermometer’ where 100 indicates ‘best imaginable health state’ and 0 indicates ‘worst imaginable health state’. The patient assesses his or her health status using both parts. For the purposes of this study, part of the Euroqol 5D questionnaire was used, namely that concerned with the prevalence of pain and anxiety among the study group [21].

### 2.2. The Geriatric Depression Scale (GDS)

The GDS was created for the assessment of depression among older people [22]. It is composed of simple answers (Yes/No). The shorter version of the test consists of 15 questions selected from the longer version. Each question is scored as 0 or 1 point. A score of 0–4 is indicative of normal; 5–8 of mild depression; 9–11 of moderate depression; and 12–15 of severe depression. The GDS is a fairly sensitive and specific test [22].

### 2.3. Handgrip Strength

The Jamar hand-held hydraulic dynamometer (Sammons Preston, Rolyon, Bolingbrook, IL, USA) was used to measure muscle strength (HGS) according to the standardized protocol [23]. The patient repeated tests 3 times with each hand. The best score was used for analysis.

### 2.4. Statistical Methods

Statistical analysis was performed using Statistica (13) software (StatSoft, Kraków, Poland).

The quantitative values are presented as means with standard deviations and qualitative variables as numbers and percentages. One-way analysis of variance (ANOVA) and the Chi^2^ test were used to compare the two groups. The logistic regression model was established to predict the probability of presence of pain and anxiety as dependent variables using the stepwise regression method, with the age, sex, body mass index (BMI), the presence of diseases, GDS score and HGS as independent variables.

A multivariate logistic regression model was constructed by employing the forward–backward stepwise selection procedure. The first model was without the GDS variable and the second was with the GDS variable.

An analysis of the comparison of the presence of pain in the group of men with HGS < 27 and with HGS ≥ 27 kg and in the group of women with HGS < 16 and with HGS ≥ 16 kg was also performed. Those cut-off points have been proposed by the European Working Group on Sarcopenia [24].

For all statistical analyses, values of *p*  <  0.05 were considered significant.

## 3. Results

### 3.1. Demographic and Clinical Data

Seventy-seven percent of the participants reported pain and sixty-five percent reported anxiety. The characteristics of the patients according to the prevalence of pain and anxiety have been presented in Table 1, Table 2, Table 3, Table 4, Table 5 and Table 6.

Subjects with pain were older, more often women, had higher BMI, higher prevalence of diabetes, heart failure, myocardial infarction, hypertension and stroke (Table 1). Subjects with anxiety were older, more often women, had higher prevalence of heart failure, hypertension and stroke (Table 2). Both pain and anxiety sufferers were characterized by higher GDS score and lower HGS as compared to the subjects without these problems (Table 1 and Table 2). Those associations were generally similar when assessed separately in women and men, especially with consistent differences for GDS and HGS (Table 3, Table 4, Table 5 and Table 6). The presence of chronic diseases was generally related to higher prevalence of pain and anxiety.

Of interest is an inverse association between the presence of musculoskeletal disorders and the manifestation of pain in women. A similar trend was observed for anxiety in women. Different associations of musculoskeletal disorders with the manifestation of pain in both sexes was confirmed with statistically significant interaction (*p* = 0.0028) between musculoskeletal disorders and sex with the manifestation of pain as a dependent variable.

### 3.2. Multivariate Regression Models

In the first multivariate logistic regression model (without the GDS), several independent predictors of pain and anxiety were identified. Statistically significant effects of female sex on the presence of pain and anxiety; higher age and BMI on the presence of pain, especially among women; chronic heart failure on the presence of pain and anxiety among both men and women; stroke on the presence of anxiety, especially among women; hypertension on the presence of pain, especially among men; and respiratory diseases and musculoskeletal disorders on the presence of pain were found.

Particular attention was directed to the effects of HGS on the presence of pain and anxiety in a multivariate design. Significant influence of HGS on the presence of pain (odds ratio [OR]  =  0.988, 95% confidence interval [CI]  =  0.980–0.995; *p*  =  0.002) (forward regression) in the entire study population was found, in addition to presence of pain among men (OR  =  0.983, 95% CI  =  0.969–0.998; *p*  =  0.022). HGS was a significant independent predictor for the presence of anxiety in the entire study population (OR  =  0.987, 95% CI  =  0.979–0.995; *p*  =  0.001), in addition to presence of anxiety among women (OR  =  0.985, 95% CI  =  0.975–0.996; *p*  =  0.005) and among men (OR  =  0.988, 95% CI  =  0.975–1.000; *p*  =  0.047). Except for the regression with pain in the entire population, all other results were the same for the forward and backward designs.

In the second multivariate logistic regression model, GDS was also included as an independent variable. GDS was selected as the most powerful (*p* < 0.001 for all analyses) independent predictor of pain or anxiety both in the entire study population and separately in women and men. Together with GDS, there were found statistically significant effects for being female on the presence of pain and anxiety; higher age on the presence of anxiety and pain, especially among women; higher BMI on the presence of pain, especially among women; chronic heart failure in all groups except anxiety among men; and hypertension on the presence of pain, especially among men.

HGS was a significant independent predictor for the presence of pain in the entire study population (OR  =  0.988, 95% CI  =  0.980–0.995; *p*  =  0.001) (forward regression), and also for the presence of anxiety in the entire study population (OR  =  0.987, 95% CI  =  0.977–0.996; *p*  =  0.007) (backward regression) and presence of anxiety among women (OR  =  0.988, 95% CI  =  0.976–1.000; *p*  =  0.049) (backward regression).

The main findings on the effects of HGS on the presence of pain and anxiety from the multivariate regression models have been shown in Table 7. Independent of other co-determinants, the contribution of HGS to the lower prevalence of pain and anxiety varied from 1.2% to 1.7% per 1 kg increase as a measure of HGS.

### 3.3. Comparison to Sarcopenia HGS Cut-Off Points

**Figure 1:**
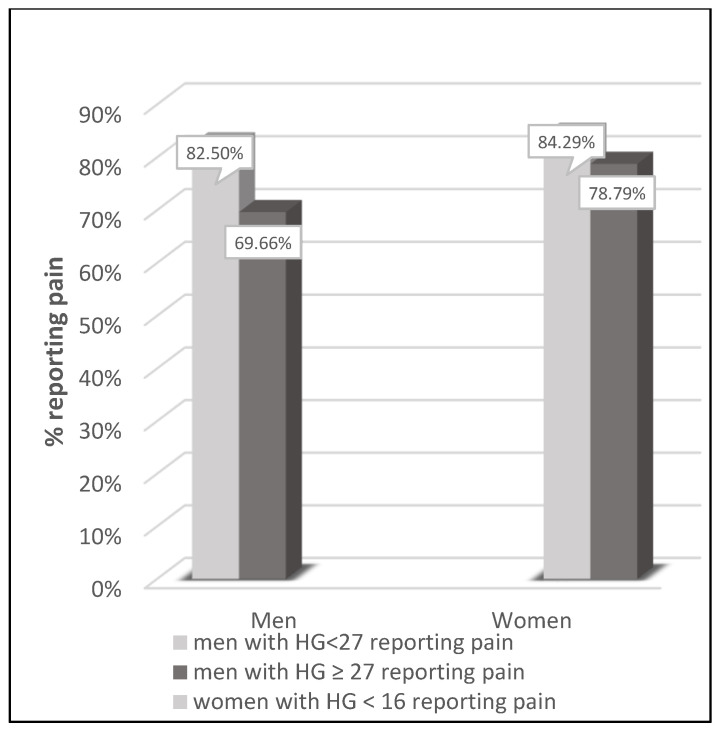
Comparison of the presence of pain by sex with a specific HGS cut-off points: in men with HGS < 27 (*n* = 40) and ≥ 27 kg (*n* = 534) and in women with HGS < 16 (*n* = 210) and HGS ≥ 16 kg (*n* = 1254). Chi^2^ score for men: *p* = 0.0858; Chi^2^ score for women: *p* = 0.0674.

**Figure 2:**
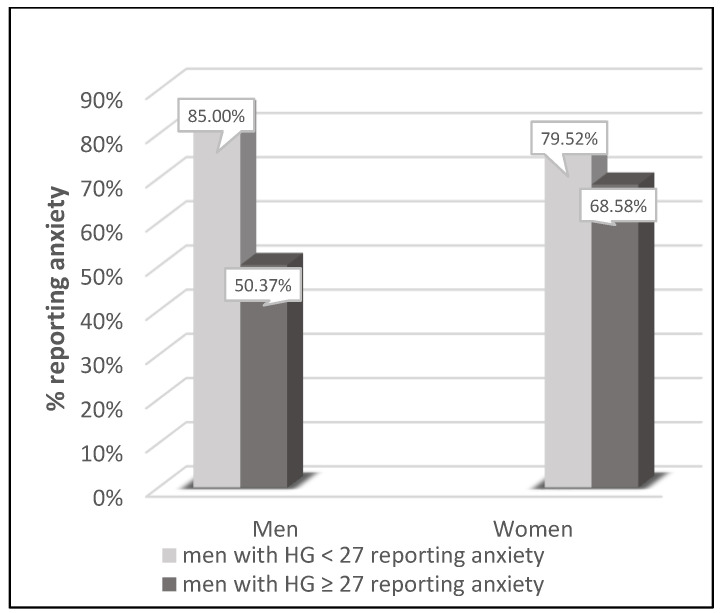
Comparison of the presence of anxiety by sex with specific HGS cut-off points: in men with HGS < 27 (*n* = 40) and ≥ 27 kg (*n* = 534) and in women with HGS < 16 (*n* = 210) and HGS ≥ 16 kg (*n* = 1254). Chi^2^ score for men: *p* < 0.001; Chi^2^ score for women: *p* = 0.0013.

Male and female groups with and without pain were compared according to sarcopenia HGS cut-off points proposed by the European Working Group on Sarcopenia (Figure 1). The prevalence of pain tended to be higher (*p* = 0.0858 for men and *p* = 0.0674 for women) in subjects fulfilling the HGS criteria for sarcopenia. Those associations were even more visible for anxiety (Figure 2). The prevalence of anxiety was higher (*p* < 0.001 for men and *p* = 0.0013 for women) in subjects fulfilling the HGS criteria for sarcopenia.

## 4. Discussion

This study analyzed the relationship between handgrip strength (HGS) and the presence of pain and anxiety among older adults. The data on this subject available in the literature are not consistent. Our study shows that both pain and anxiety are very common in the study population and that HGS influences the presence of pain and anxiety among older adults independent of age, sex and concomitant chronic diseases. This relationship is generally similar in males and females, but the co-contribution of concomitant disorders may differ between the two sexes.

The prevalence of pain and anxiety increases with age [2,3]. Both pain and anxiety are associated with decreased functioning, poor mental health and have a negative impact on quality of life for older people [3,25,26]. Chronic pain and anxiety also increase health care costs [3,26]. HGS is a simple, quick and reliable method of assessing maximum voluntary compressive force. This measurement is useful for assessing qualitative and functional aspects of muscle strength, but also for evaluating nutritional status and general health. In addition, HGS is associated with many chronic diseases [27].

Recent data have shown that HGS can be an independent predictor of quality of life in a variety of disease settings, from arthritis to chronic liver disease and depression [28]. Lower HGS was significantly associated with prevalence of pain, anxiety and poor quality of life in cancer survivors [28,29]. Pain intensity and HGS were dysfunctions affecting upper limb disability in women with bilateral idiopathic carpal tunnel syndrome [15], and carpal tunnel syndrome patients with low levels of education showed reduced HGS and more catastrophic thinking [16]. The results of the study by Daliri et al. [17] show that anxiety correlates with pain and lower HGS in patients with cervical radiculopathy. Furthermore, this correlation is almost similar in patients with carpal tunnel syndrome. Disability in patients with carpal tunnel syndrome as well as cervical radiculopathy is associated with anxiety, depression and catastrophic thinking. The authors suggest that further research should determine whether psychological distress causes more disability or the reverse [17]. In a cross-sectional study of 439 women with fibromyalgia, lower physical performance assessed through the Senior Fitness Test battery and HGS test was associated with higher levels of anxiety [30]. In women with early rheumatoid arthritis HGS was found to be significantly reduced in those with widespread pain as compared to women without widespread pain [31]. The effects of preoperative pain, anxiety and depression on arm, shoulder and hand disability; quality of life; HGS strength and range of motion in the first year after salvage wrist surgery were studied [18]. Negative effects of pain and anxiety on patient-reported outcomes were found. Preoperative pain or tendency to anxiety had a strong negative effect on postoperative disability. Anxiety also resulted in lower postoperative quality of life, while pain had a negative effect on HGS [18]. In contrast, severe fatigue in people with rheumatoid arthritis was associated more with self-rated health, pain and anxiety/depression rather than with physical capacity tests (lower limb function, HGS and aerobic capacity) [32].

Similar to with specific diseases, several general population studies indicated an association between HGS and mental health. Korean men and women with low HGS had poor mobility and pain or discomfort on the Euroqol-5D scale [33]. In middle-aged Japanese women, low grip strength and insomnia were independently associated with pain symptoms. The authors suggested that treating insomnia in these women may offset muscle and joint pain and thus improve HGS, or treating pain may reduce insomnia in addition to improving HGS [34]. In 3952 subjects aged ≥50 years, a one-standard-deviation increase in HGS was associated with 12.1% lower odds of prevalent Generalized Anxiety Disorder (GAD), and middle- and high-strength tertiles were associated with 27.3% and 23.1% lower odds, respectively [35]. In a 7-year prospective cohort study of 152,978 UK Biobank participants, an exercise test and dynamometer were used to measure cardiorespiratory fitness and HGS, respectively [36]. Patient Health Questionnaire-9 and Generalised Anxiety Disorder-7 scales estimated the incidence of common mental disorders at follow-up. Low and medium HGS were associated with 1.381 and 1.116 higher odds of common mental disorder compared to high HGS. Individuals in the lowest group for both cardiorespiratory fitness and HGS had 1.981 higher odds of depression and 1.599 higher odds of anxiety, compared to a high level of fitness group [36]. In another analysis from the UK Biobank prospective cohort study, of the 162,167 participants included, 5462 (3.4%) developed depression and 6614 (4.1%) anxiety, over a median follow-up period of 10.0 years. In the fully adjusted model, a 5 kg lower HGS rating was associated with a 7% and 8% higher risk of depression and anxiety, respectively. The authors recommend that future research should assess if resistance training aimed at increasing HGS can prevent the occurrence of mental health conditions [37].

Relatively few studies assessed the relationship of HGS to the prevalence of pain or anxiety in older populations. Imai et al. assessed the frailty and chronic pain of 107 older adults. The prevalence of chronic pain with pre-frailty was high, and chronic pain and pre-frailty were strongly related [26]. A recent meta-analysis demonstrated a relationship between low muscle strength and intensified depressive symptoms in older populations [38]. In contrast, in 173 institutionalized older individuals, there were no differences in HGS values between the groups with and without chronic pain [39].

Our data show that both pain and anxiety are very common in the older population, especially in women. Presence of symptoms of depression (GDS) was selected as the most powerful independent predictor of pain or anxiety, both in the entire study population and separately in women and men. Higher HGS was related to the lower presence of pain and anxiety among older adults independent of age, GDS and concomitant chronic diseases. In the fully adjusted model, a HGS rating higher than 1kg was associated with 1.2% and 1.3% lower probability of the presence of pain and anxiety, respectively. These data seem comparable to the analysis from the UK Biobank prospective cohort [37]. The association of HGS to the prevalence of pain and anxiety was apparently similar in males and females. Nevertheless, the co-contribution of concomitant disorders may differ between the two sexes. While the presence of chronic diseases was generally related to higher prevalence of pain and anxiety, an inverse association of the presence of musculoskeletal disorders to the manifestation of pain, with a similar trend for anxiety, was observed in women. This phenomenon may be explained by the fact that women with musculoskeletal disorders were almost three years younger (*p* < 0.001) as compared to women without those diseases. Therefore, it seems that younger women are more prone to suffer from musculoskeletal diseases, while with age this problem becomes less troublesome.

Our study has some limitations. Its cross-sectional character excludes any firm causality statements. In addition, the study was conducted in a central European population, and results may be different in other cultures. Nevertheless, the key strengths of this study are the careful recruitment procedures and the large study population.

## 5. Conclusions

A significant relationship between low HGS and the presence of pain and anxiety in the study population was found. Maintenance of high HGS may be considered an important protective factor against feelings of pain and anxiety, independent of age, depression and concomitant diseases. Future research should prospectively assess reciprocal associations between HGS and pain or anxiety and whether improvement of HGS would alleviate psychological dysfunction in older adults. It seems that this would also be helpful for the geriatric healthcare system.

## Figures and Tables

**Table 1:** Characteristics of pain prevalence among study patients.

		Pain	
Variable	Yes (*n* = 1570)	No (*n* = 468)	*p*-Value
Age [years]	74.53 ± 8.47	72.16 ± 8.13	≤0.001 ^a^
Men [*n* (%)]	405 (25.8)	169 (36.1)	≤0.001 ^b^
BMI [kg/m^2^]	27.46 ± 4.80	26.94 ± 4.42	≤0.05 ^a^
Diabetes [*n* (%)]	313 (19.9)	74 (15.8)	≤0.05 ^b^
Respiratory diseases [*n* (%)]	201 (12.8)	50 (10.7)	ns ^b^
Musculoskeletal disorders [*n* (%)]	326 (20.8)	102 (21.8)	ns ^b^
Chronic heart failure [*n* (%)]	626 (39.9)	103 (22)	≤0.001 ^b^
Myocardial infarction [*n* (%)]	159 (10.1)	30 (6.4)	≤0.05 ^b^
Hypertension [*n* (%)]	1072 (68.3)	267 (57)	≤0.001 ^b^
Stroke [*n* (%)]	193 (12.3)	36 (7.7)	≤0.01 ^b^
Cancer [*n* (%)]	131 (8.3)	34 (7.3)	ns ^b^
GDS	5.03 ± 3.76	3.05 ± 2.93	≤0.001 ^a^
HGS [kg]	30.11 ± 14.23	34.21 ± 14.94	≤0.001 ^a^

The quantitative values are presented as mean ± SD and as number and percentage. ^a^ Analysis of variance (ANOVA) test; ^b^ Chi^2^ test. Abbreviations: BMI—body mass index, GDS—Geriatric Depression Scale, HGS—handgrip strength.

**Table 2:** Characteristics of anxiety prevalence among study patients.

		Anxiety	
Variable	Yes (*n* = 1330)	No (*n* = 708)	*p*-Value
Age [years]	74.48 ± 8.41	73.06 ± 8.45	≤0.001 ^a^
Men [*n* (%)]	303 (22.8)	271 (38.3)	≤0.001 ^b^
BMI [kg/m^2^]	27.35 ± 4.87	27.32 ± 4.42	ns ^a^
Diabetes [*n* (%)]	269 (20.2)	118 (16.7)	ns ^b^
Respiratory diseases [*n* (%)]	171 (12.8)	80 (11.3)	ns ^b^
Musculoskeletal disorders [*n* (%)]	280 (21)	148 (20.9)	ns ^b^
Chronic heart failure [*n* (%)]	544 (40.9)	185 (26.1)	≤0.001 ^b^
Myocardial infarction [*n* (%)]	134 (10.1)	55 (7.8)	ns ^b^
Hypertension [*n* (%)]	902 (67.8)	437 (61.7)	≤0.01 ^b^
Stroke [*n* (%)]	172 (12.9)	57 (8)	≤0.001 ^b^
Cancer [*n* (%)]	111 (8.3)	54 (7.6)	ns ^b^
GDS	5.6 ± 3.71	2.65 ± 2.73	≤0.001 ^a^
HGS [kg]	29.21 ± 13.7	34.52 ± 15.32	≤0.001 ^a^

The quantitative values are presented as mean ± SD and as number and percentage. ^a^ Analysis of variance (ANOVA) test; ^b^ Chi^2^ test. Abbreviations: BMI—body mass index, GDS—Geriatric Depression Scale, HGS—handgrip strength, ns—not significant.

**Table 3:** Characteristics of pain prevalence among women.

		Pain among Women	
Variable	Yes (*n* = 1165)	No (*n* = 299)	*p*-Value
Age [years]	75.29 ± 8.45	72.92 ± 8.27	≤0.001 ^a^
BMI [kg/m^2^]	27.51 ± 4.94	26.63 ± 4.52	≤0.01 ^a^
Diabetes [*n* (%)]	226 (19.4)	37 (12.37)	≤0.01 ^b^
Respiratory diseases [*n* (%)]	127 (10.9)	36 (12.04)	ns ^b^
Musculoskeletal disorders [*n* (%)]	241 (20.7)	79 (26.42)	≤0.05 ^b^
Chronic heart failure [*n* (%)]	482 (41.4)	75 (25.08)	≤0.001 ^b^
Myocardial infarction [*n* (%)]	98 (8.4)	18 (6.02)	ns ^b^
Hypertension [*n* (%)]	793 (68.1)	184 (61.54)	≤0.05 ^b^
Stroke [*n* (%)]	138 (11.8)	23 (7.69)	≤0.05 ^b^
Cancer [*n* (%)]	101 (8.7)	24 (8.03)	ns ^b^
GDS	5.20 ± 3.77	3.33 ± 3.09	≤0.001 ^a^
HGS [kg]	25.31 ± 10.69	27.28 ± 11.41	≤0.01 ^a^

The quantitative values are presented as mean ± SD and as number and percentage. ^a^ Analysis of variance (ANOVA) test; ^b^ Chi^2^ test. Abbreviations: BMI—body mass index, GDS—Geriatric Depression Scale, HGS—handgrip strength, ns—not significant.

**Table 4:** Characteristics of pain prevalence among men.

		Pain among Men	
Variable	Yes (*n* = 405)	No (*n* = 169)	*p*-Value
Age [years]	72.34 ± 8.14	70.82 ± 7.71	≤0.05 ^a^
BMI [kg/m^2^]	27.29 ± 4.38	27.49 ± 4.2	ns ^a^
Diabetes [*n* (%)]	87 (21.5)	37 (21.89)	ns ^b^
Respiratory diseases [*n* (%)]	74 (18.3)	14 (8.28)	≤0.01 ^b^
Musculoskeletal disorders [*n* (%)]	85 (21)	23 (13.61)	≤0.05 ^b^
Chronic heart failure [*n* (%)]	144 (35.6)	28 (16.57)	≤0.001 ^b^
Myocardial infarction [*n* (%)]	61 (15.1)	12 (7.1)	≤0.01 ^b^
Hypertension [*n* (%)]	279 (68.9)	83 (49.11)	≤0.001 ^b^
Stroke [*n* (%)]	55 (13.6)	13 (7.69)	≤0.05 ^b^
Cancer [*n* (%)]	30 (7.4)	10 (5.92)	ns ^b^
GDS	4.54 ± 3.7	2.56 ± 2.56	≤0.001 ^a^
HGS [kg]	43.91 ± 14.17	46.45 ± 12.37	≤0.05 ^a^

The quantitative values are presented as mean ± SD and as number and percentage. ^a^ Analysis of variance (ANOVA) test; ^b^ Chi^2^ test. Abbreviations: BMI—body mass index, GDS—Geriatric Depression Scale, HGS—handgrip strength, ns—not significant.

**Table 5:** Characteristics of anxiety prevalence among women.

		Anxiety among Women	
Variable	Yes (*n* = 1027)	No (*n* = 437)	*p*-Value
Age [years]	75.17 ± 8.46	73.96 ± 8.42	≤0.05 ^a^
BMI [kg/m^2^]	27.43 ± 4.98	27.11 ± 4.6	ns ^a^
Diabetes [*n* (%)]	201 (19.6)	62 (14.19)	≤0.05 ^b^
Respiratory diseases [*n* (%)]	115 (11.2)	48 (10.98)	ns ^b^
Musculoskeletal disorders [*n* (%)]	219 (21.3)	101 (23.11)	ns ^b^
Chronic heart failure [*n* (%)]	433 (42.2)	124 (28.38)	≤0.001 ^b^
Myocardial infarction [*n* (%)]	86 (8.4)	30 (6.86)	ns ^b^
Hypertension [*n* (%)]	700 (68.2)	277 (63.39)	ns ^b^
Stroke [*n* (%)]	128 (12.5)	33 (7.55)	≤0.01 ^b^
Cancer [*n* (%)]	92 (9)	33 (7.55)	ns ^b^
GDS	5.66 ± 3.7	2.82 ± 2.91	≤0.001 ^a^
HGS [kg]	25.06 ± 10.32	27.26 ± 11.91	≤0.001 ^a^

The quantitative values are presented as mean ± SD and as number and percentage. ^a^ Analysis of variance (ANOVA) test; ^b^ Chi^2^ test. Abbreviations: BMI—body mass index, GDS—Geriatric Depression Scale, HGS—handgrip strength, ns—not significant.

**Table 6:** Characteristics of anxiety prevalence among men.

		Anxiety among Men	
Variable	Yes (*n* = 1027)	No (*n* = 437)	*p*-Value
Age [years]	72.16 ± 7.81	71.59 ± 8.29	ns ^a^
BMI [kg/m^2^]	27.08 ± 4.48	27.65 ± 4.13	ns ^a^
Diabetes [*n* (%)]	68 (22.4)	56 (20.66)	ns ^b^
Respiratory diseases [*n* (%)]	56 (18.5)	32 (11.81)	≤0.05 ^b^
Musculoskeletal disorders [*n* (%)]	61 (20.1)	47 (17.34)	ns ^b^
Chronic heart failure [*n* (%)]	111 (36.6)	61 (22.51)	≤0.001 ^b^
Myocardial infarction [*n* (%)]	48 (15.8)	25 (9.23)	≤0.05 ^b^
Hypertension [*n* (%)]	202 (66.7)	160 (59.04)	ns ^b^
Stroke [*n* (%)]	44 (14.5)	24 (8.86)	≤0.05 ^b^
Cancer [*n* (%)]	19 (6.3)	21 (7.75)	ns ^b^
GDS	5.39 ± 3.75	2.36 ± 2.39	≤0.001 ^a^
HGS [kg]	43.26 ± 14.38	46.22 ± 12.75	≤0.01 ^a^

The quantitative values are presented as mean ± SD and as number and percentage. ^a^ Analysis of variance (ANOVA) test; ^b^ Chi^2^ test. Abbreviations: BMI—body mass index, GDS—Geriatric Depression Scale, HGS—handgrip strength, ns—not significant.

**Table 7:** Independent of other co-determinants, the contribution of HGS (increase in HGS per 1 kg) to the lower prevalence of pain and anxiety among study patients.

	Multivariate Logistic Regression Model without GDS			Multivariate Logistic Regression Model with GDS		
	Study Population	Women	Men	Study Population	Women	Men
Pain	↓1.2%	-	↓1.7%	↓1.2%	-	-
Anxiety	↓1.5%	↓1.3%	↓1.2%	↓1.3%	↓1.2%	-

GDS—Geriatric Depression Scale, HGS—handgrip strength.

## Data Availability

Data will be available on request from the corresponding author.
